# Supplementary material for: Brain Iron as a Surrogate Biomarker of Pathological TDP-43 Identifies Brain Region-Specific Signatures in Ageing, Alzheimer’s Disease and Amyotrophic Lateral Sclerosis
Source: bioRxiv. 2025 Oct 3:2025.10.02.680028. Preprint. [Version 1] doi: 10.1101/2025.10.02.680028 (PMC12621661; doi:10.1101/2025.10.02.680028)
Supplement: 1 [file NIHPP2025.10.02.680028V1-supplement-1.pdf]

bioRxiv preprint doi: https://doi.org/10.1101/2025.10.02.680028; this version posted October 3, 2025. The copyright holder for this preprint (which was not certified by peer review) is the author/funder, who has granted bioRxiv a license to display the preprint in perpetuity. It is made available under aCC-BY 4.0 International license.

Supplementary Information

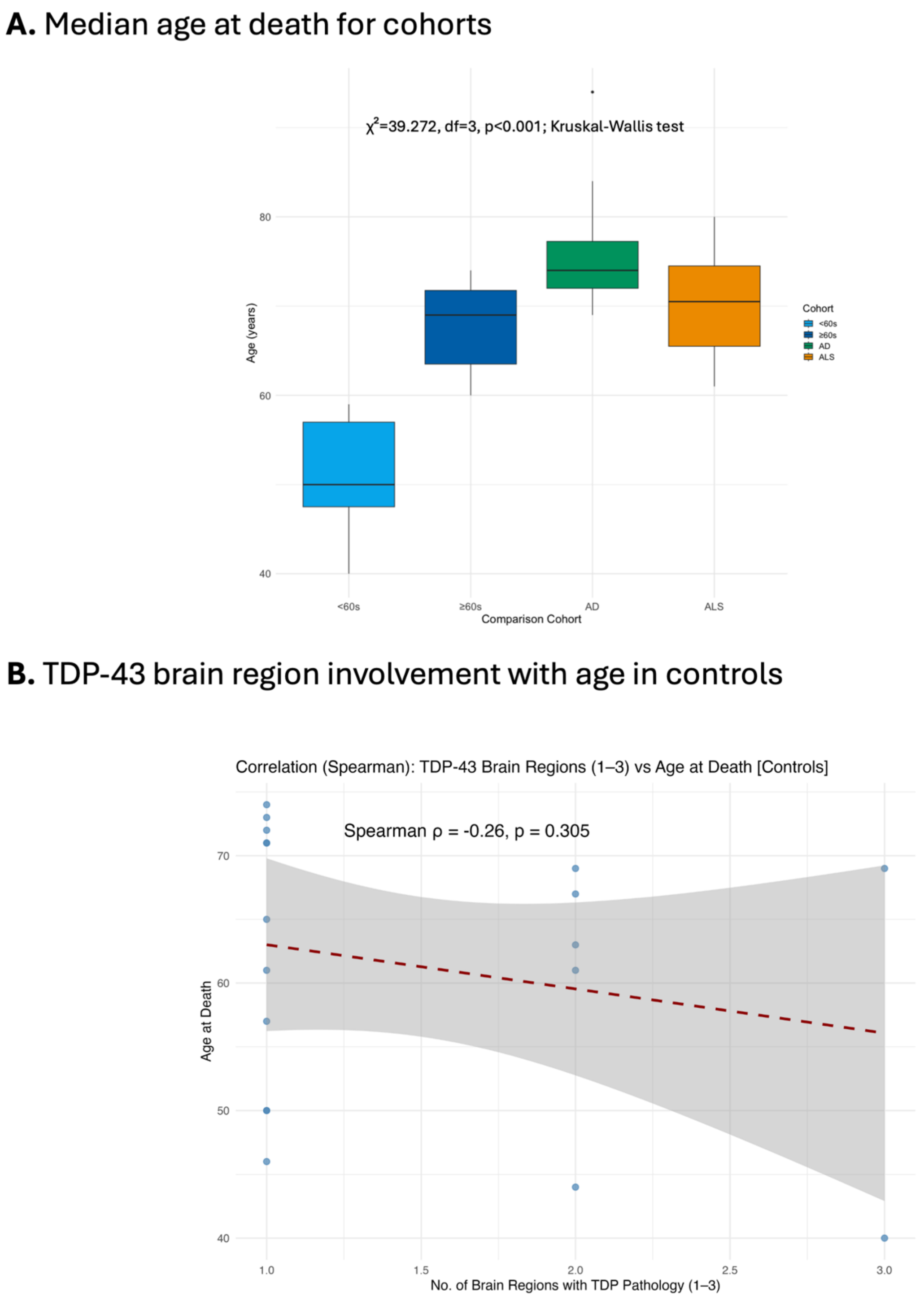

Supplementary Figure 1. A, Median age at death boxplots for each of the four study cohorts (<60s, ≥60s, ALS and AD) (see also table 2). **B,** TDP-43 brain region involvement with age in ageing controls.

bioRxiv preprint doi: https://doi.org/10.1101/2025.10.02.680028; this version posted October 3, 2025. The copyright holder for this preprint (which was not certified by peer review) is the author/funder, who has granted bioRxiv a license to display the preprint in perpetuity. It is made available under aCC-BY 4.0 International license.

Additional analysis

Ferritin level differences are brain region-specific in ageing and disease

Because brain ferritin accumulated iron can be measured by MRI, we need to understand brain region differences in ageing and disease. In addition to the analyses comparing ferritin levels across all four cohorts (<60s, ≥60s, AD, ALS) together, we analysed ageing- and disease-comparable groups separately here, but without significant differences in results.

To do these analyses we tested brain-region specific ageing-related differences in ferritin levels between <60s and ≥60s ("Ageing Comparison), and disease-related differences in ferritin levels between AD, ALS and the age-matched ≥60s cohort (Disease Comparison).

No evidence for non-normality in ferritin level data was uncovered for <60s, ≥60s, AD or ALS cohort by Shapiro-Wilk tests, however inequality of variances amongst groups in the "Ageing comparison" between <60s and ≥60s (F=8.547, df=1,30, p=0.007; Levene's test), and in the "Disease Comparison" between AD, ALS and ≥60s (F=5.1855, df=2,29, p=0.012; Levene's test), informed our non-parametric approach here.

Hippocampus ferritin is an AD-specific marker of disease

Amygdala ferritin levels similar across ageing and disease

Amygdala median ferritin levels were higher in ≥60s than in <60s but this difference was not significant (W=86, p=0.091; Wilcoxon rank sum test) (**Suppl. Fig. 2**). No significant difference in amygdala ferritin levels between AD, ALS or age-matched ≥60s (χ^2= 2.059, df=2, p= 0.357; Kruskal-Wallis rank sum test) was revealed (**Suppl. Fig. 2).**

Higher hippocampus ferritin levels is a disease-specific signature of AD

Hippocampus median ferritin levels were higher in ≥60s than in <60s but this difference was not significant (W=151, p=0.305; Wilcoxon rank sum test) (**Suppl. Fig. 2**). However, hippocampus ferritin levels were significantly different in the disease comparison between AD, ALS, and age-matched ≥60s (χ^2= 9.502, df=2, p= 0.009; Kruskal-Wallis rank sum test) (**Suppl. Fig. 2**). Here, for AD significantly higher hippocampus ferritin levels were increased compared to age-matched (i.e. ≥60s) controls (z= 3.08, p= 0.006; Dunn's test).

Hippocampus ferritin levels in ALS were lower but not significantly different to AD (z= 1.54, p= 0.372; Dunn's test), or significantly different compared to age-matched ≥60s (z=1.45 =, p= 0.455; Dunn's test) (**Suppl. Fig. 2**).

Higher frontal cortex ferritin levels in disease are likely a function of ageing

Frontal cortex median ferritin levels were higher in ≥60s than in <60s (W=180, p=0.030; Wilcoxon rank sum test) (**Suppl. Fig. 2**), however but not significantly different in the disease comparison between AD, ALS or age-matched ≥60s cohorts (χ^2=4.094, df=2, p= 0.129; Kruskal-Wallis rank sum test).

bioRxiv preprint doi: https://doi.org/10.1101/2025.10.02.680028; this version posted October 3, 2025. The copyright holder for this preprint (which was not certified by peer review) is the author/funder, who has granted bioRxiv a license to display the preprint in perpetuity. It is made available under aCC-BY 4.0 International license.

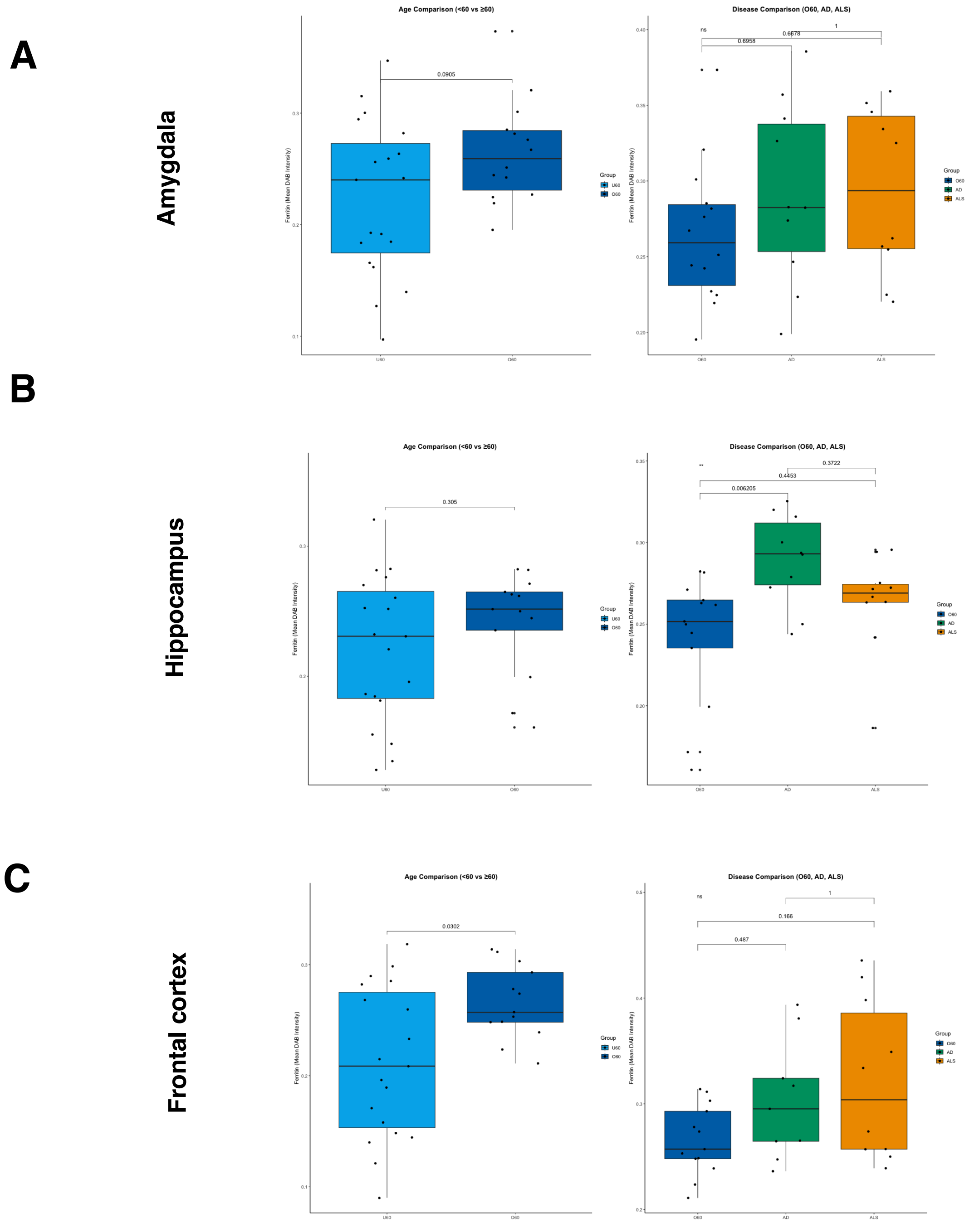

Supplementary Figure 2: Increased hippocampus ferritin levels are a disease-specific signature of AD. For amygdala (A), hippocampus (B) and frontal cortex (C), median ferritin levels were statistically compared between <60s and ≥60s (“Ageing Comparison), and AD, ALS and the age-matched ≥60s cohort (Disease Comparison) using Wilcoxon rank sum tests and Kruskal-Wallis rank sum tests, respectively with p-values for group comparison presented with significance bars.
